# Hematopoietic (stem) cells—The elixir of life?

**DOI:** 10.1002/1873-3468.70215

**Published:** 2025-12-04

**Authors:** Emilie L. Cerezo, Jonah Anderson, Emilie Dinh Vedrenne, Noël Yeh Martín, Jette Lengefeld

**Affiliations:** ^1^ Helsinki Institute of Life Science, HiLIFE, Institute of Biotechnology, Faculty of Biological and Environmental Sciences University of Helsinki Finland; ^2^ Center for Hematology and Regenerative Medicine, Department of Medicine Huddinge Karolinska Institutet Stockholm Sweden

**Keywords:** blood system, health span, hematopoietic stem cells, organismal aging, rejuvenation

## Abstract

The long lifespan of humans is often not matched with health span. Thus, there is a need for rejuvenation strategies. Here, we first discuss the evolutionary benefits of the long human lifespan, particularly when coupled with an extended health span. We then highlight the importance of understanding the complexity of aging before interfering with it. This raises the question of the optimal target for rejuvenation. We propose the blood system and hematopoietic stem cells (HSCs). Their decline is associated with dysfunction and disease in other organs, crystallizing them as a central player in organismal aging. We present rejuvenation strategies targeting the hematopoietic system, especially HSCs, and explore their systemic benefits. Overall, we summarize the potential of the blood system to reverse aging.

**Impact statement**

There is a current need to reduce the economic burden caused by aging‐related diseases. In this perspective article, we discuss the evidence that supports that rejuvenating or delaying aging of the blood system has a beneficial and systemic impact on human health.

## Graphical abstract

The aging of HSCs (hematopoietic stem cells) and the blood system leads to the decline of other organs. Rejuvenating aged HSCs improves the function of the blood system, slowing the aging of the heart, kidney, brain, and liver, and the occurrence of age‐related diseases. The blood system is therefore a promising target to improve organismal health during aging.

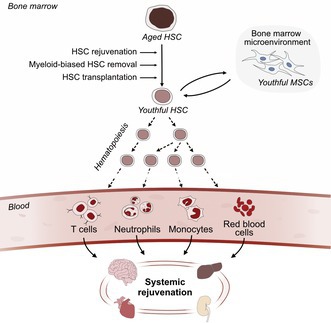

## Abbreviations

**aHSC**, autologous hematopoietic stem cell

**AIDS**, acquired immunodeficiency syndrome

**Aβ**, amyloid beta

***C. elegans***, *Caenorhabditis elegans*

**CAR**, chimeric antigen receptor

**CNS**, central nervous system

**CR**, calorie restriction

**DNA**, deoxyribonucleic acid

**eccDNA**, extrachromosomal circular DNA

**HGPS**, Hutchinson‐Gilford progeria syndrome

**HIV**, human immunodeficiency viruses

**HP**, heterochronic parabiosis

**HSC**, hematopoietic stem cell

**iPSC**, induced pluripotent stem cell

**MSC**, mesenchymal stem cell

**mTOR**, mammalian target of rapamycin

**NAD** ^**+**^, nicotinamide adenine dinucleotide

**NK**, natural killer

**RBC**, red blood cell

**ROS**, reactive oxygen species

**TPE**, therapeutic plasma exchange

The quest for longevity is an old tale [1]. Recently, an unmatched interest in anti‐aging and rejuvenation strategies has emerged. From skincare products to anti‐aging food, a flood of new products claims their age‐defying benefits [2]. Longevity and rejuvenation have become a global business market [3], valued at more than $42 billion in 2024 [4]. Current global annual investment in geroscience exceeds $10 billion, combining public, philanthropic, and venture capital sources [5].

Given this interest in rejuvenation, we discuss the importance of understanding aging before reversing it. We then identify the blood system as a key rejuvenation target due to its crucial role in organismal aging.

## What is aging?

From the moment we are born, several processes take place over time, including development, adaptation, and functional decline. Aging refers to the latter and is often defined as time‐dependent deterioration of physiological functions [6]. Aging is driven by aging factors or hallmarks, which (i) manifest during aging, (ii) when induced accelerate aging, (iii) and when removed slow down aging [7]. The list included the following: cellular senescence, mitochondrial dysfunction, stem cell exhaustion, telomere attrition, altered intercellular communication, deregulated nutrient sensing, loss of proteostasis, genomic instability, disabled macroautophagy, chronic inflammation, dysbiosis, epigenetic alterations, and other emerging aging factors, such as cellular enlargement [7, 8, 9, 10, 11, 12]. The aging hallmarks provide a starting point to test interventions with rejuvenation potential. However, the aging process remains not fully understood and the number of aging hallmarks is continuously expanding.

## Why rejuvenate?

We live longer than ever before [13]. However, health span—the period without chronic diseases and disabilities—does not match the extending lifespan, which increases the incidence of age‐related diseases and the associated socio‐economic burden [14, 15]. Reversing or slowing aging would delay the onset of age‐related diseases like cardiovascular, neurodegenerative, metabolic, and hematological disorders [13, 16, 17, 18, 19]. This approach has been proposed to be economically more beneficial than treating diseases individually [20].

Before reversing aging, it is important to first ask why it exists in humans in the first place. Humans have a comparatively long lifespan of ~80 years with 122 years as the oldest recorded [13, 21]. What are the reasons for this long lifespan? Theodosius Dobzhansky said ‘Nothing makes sense in biology except in the light of evolution’ [22]. Evolutionary aging theories suggest that natural selection acts mostly at younger ages when reproduction is high and mutations are passed onto the next generation. Thus, genes that cause decline at old age are less likely to be eliminated by natural selection [23, 24, 25]. For humans, evolutionary pressure may even continue after the reproductive phase. One observation supporting this is known as the grandmother effect, where the presence of grandmothers is associated with increased survival and reproductive success of their grandchildren [26, 27, 28, 29]. This would provide an explanation of why women live long after their menopause. The grandmother effect is only observed so far in humans, orcas, and elephants [30, 31, 32] and is most likely not the only factor connecting reproductive strategies with lifespan. Interestingly, some gene variant alleles present uniquely in humans have been proposed to protect against cognitive decline at old age. An interpretation is that elderly people carrying these variant alleles maintained their cognitive functions longer, which would similarly allow them to support their reproductive offspring [33]. Thus, improving the health and lifespan of postreproductive individuals in our society is expected to support the fitness of younger ones. Furthermore, in many organisms, the number of cortical neurons correlates with both total lifespan and length of the developmental period [34]. The long lifespan and postnatal development of humans may allow for more developed brains capable of complex social interactions and the creation of advanced tools, which both positively impact lifespan [35]. Together, these observations suggest that the long lifespan of humans evolved to allow complex brain development and the presence of elderly people to ensure the survival of their genes in the younger generations.

Considering the advantages of a long lifespan, why is lifespan limited at all? An interesting discovery was that certain gene manipulations extend lifespan [36, 37], for example the *daf‐2* gene in *C. elegans* [36, 38, 39] and potentially its human homolog [40]. Originally, this raised the question of whether genes exist that limit lifespan, which would imply the existence of selective pressure against increased lifespan. However, *daf‐2* has pleiotropic functions; for example, it is important for proper development [41]. Thus, its function in early life likely provides a greater evolutionary benefit than the cost of limiting lifespan later in life (known as antagonistic pleiotropy [42]). Nevertheless, genes that limit lifespan are interesting as targets for treatments aiming to increase lifespan after the reproductive period [43].

One important point is that extending lifespan seems to only benefit an organism if health span is extended at the same time. Indeed, extending lifespan reduces the resistance to natural stresses in several model organisms [44] and increases time spent in a frail state in *C. elegans* [39] and humans [14, 15, 45]. Health span in humans is restricted by the limited regenerative ability of organs like the heart, spinal cord, and brain [46] and the aging‐dependent decline of regeneration in organs, such as bone marrow, liver, intestine, and skeletal muscle [47, 48, 49]. These observations raise the question of why we did not evolve regenerative potential that persists at old age in all organs. One interpretation is that regeneration increases cancer risk [50, 51], which may shorten lifespan even more than decay from aging. Thus, prolonging health span via increased regeneration requires balancing to prevent tumor formation. Altogether, these observations suggest that there are evolutionary benefits of a healthy and long lifespan in humans.

## Compensatory adaptations during old age

Over time, several processes take place, including adaptations to decay from aging [52]. Examples of time‐dependent adaptation were provided by research in *Saccaromyces cerevisiae* (budding yeast), a powerful model system for eukaryotic aging [53]: Old yeast cells grow and adapt better than younger ones when nutritional conditions change [54, 55]. This rapid adaptation may result from the time‐dependent accumulation of stress protectant molecules and specific extrachromosomal circular DNA (eccDNA), which provides a reservoir of heterogeneous molecular material [56, 57]. eccDNA also exists in human cells, opening the possibility that these adaptive mechanisms are conserved. They are proposed to provide adaptive advantages in the cancer context [56, 58, 59, 60, 61]. Furthermore, low‐level activation of stress responses upon age‐associated damage also improves resistance to external stresses (hormesis) [62, 63]. Importantly, these observations suggest that not everything occurring with old age directly leads to decay but can instead be a compensatory adaptation to temporarily maintain functions.

There are more examples of processes originally thought to exclusively drive aging, which then turned out to (also) support physiological functions:

- *Amyloid beta (Aβ) plaques* accumulate during aging and are consistently observed in postmortem brains with Alzheimer's disease. Hence, Aβ plaques were first considered as a main pathogenic driver of Alzheimer's [64]. Now, models suggest that Aβ plaques may be neuroprotective as they sequester toxic Aβ forms, thereby preventing the formation of amyloid pores [64, 65, 66].
- *Somatic mutations* accumulate during aging and are the root cause of cancer. While they were initially assumed to drive aging, this is now under debate [12, 67, 68]. Most likely, somatic mutations are associated with gradual functional decline and increased vulnerability to disease; however, there are exceptions which restore organ function. For example, the germline variant *COL17A1* is associated with skin disease. Somatic mutations in this gene can result in a selection advantage leading to an improvement of symptoms [69]. Similar observations were made for germline mutations of Mendelian hematopoietic diseases [70] and Hutchinson–Gilford progeria [71]. Furthermore, somatic mutations have been reported to confer cancer protection in the epithelium and promote liver regeneration [72, 73]. Exploring the mutational landscape of centenarians—persons ≥100 years—may uncover beneficial somatic mutations for human longevity [74, 75]. Somatic mutations also accumulate in the blood system and are present in around 15% of 70‐year‐olds harboring mutated clones. These mutations initially improve blood‐building capacity; however, ultimately, all clonal expansions in the blood are associated with increased risk of hematological malignancy [76, 77].
- *Senescent cells* are permanently arrested in the cell cycle, accumulate during aging and are a major component of aging dysfunction [78, 79, 80]. Indeed, transplanting senescent cells into mice drives age‐related diseases [81, 82, 83, 84]. However, it has been revealed that senescence also supports physiological functions like tissue remodeling during embryonic development, wound healing, removal of premalignant cells, and hemostasis (the process of bleeding cessation) [85, 86, 87, 88, 89, 90, 91, 92]. Indeed, certain senolytics are associated with thrombocytopenia that impairs hemostasis [93, 94, 95]. Thus, it would be optimal to pharmacologically distinguish between pathological and physiological senescence.
- *Reactive oxygen species* (ROS) accumulate over time. However, increasing evidence indicates no direct correlation between ROS accumulation and accelerated aging. The physiological function of ROS production is proposed to contribute to intracellular signaling rather than stochastic macromolecular damage [96]. In agreement with this, ROS generation governs the metabolic benefits of physical exercise in humans via transcriptional reprogramming [97].

Overall, these examples illustrate the importance of first understanding the mechanisms occurring during old age before targeting them. Aspects of aging itself have been proposed to maintain physiological functions. For example, while aging is the most important risk factor for cancer [98, 99], it also has been suggested to be cancer protective [99, 100]. Many cells decline in proliferative potential during aging [101, 102], while cancerous cells are characterized by increased proliferation. Telomere attrition causes cellular aging and is often counteracted by overexpression of telomerase in cancer cells [103]. Expressing oncogenes can induce senescence in cells, while drivers of senescence, such as p21, p16, and p53, are often dysfunctional in tumors [104, 105, 106, 107]. However, the picture is more complex as some senescent cells can exit their cell cycle arrest and drive cancer relapse [108, 109].

Another cancer protective example driven by aging is provided by Hutchinson–Gilford progeria syndrome (HGPS), which is caused by progerin generation. Individuals affected by the syndrome experience premature aging and display increased levels of DNA damage [110, 111]. However, this increased genetic instability does not correlate with a higher cancer risk [110, 112] due to the protective effect of progerin [113]. Upon aging, progerin also accumulates in normal tissues suggesting that its protective properties may also occur in physiological conditions [114, 115, 116]. Thus, has aging evolved to suppress cancer? This will remain a hard question to disentangle since another interpretation reverses this viewpoint: mechanisms evolved to suppress cancer until they fail due to aging [117]. Regardless, aging and cancer are linked in humans and any attempt to rejuvenate must take care that the intervention does not lead to malignancy. Taken together, not everything that occurs during the later years of an organism can be taken at face value as a direct driver of aging. This highlights the need for caution when aiming to intervene in aging mechanisms to rejuvenate.

## What to rejuvenate? The blood system as a star(t) ☆

To effectively rejuvenate, we should consider that aging is a complex process that manifests differently across individuals of the same chronological age. Moreover, in the same individual, aging rates vary across tissues, organs [118, 119] and cell type populations [120, 121, 122], thereby influencing the development of distinct age‐related diseases and associated comorbidities [118, 123]. An optimal target of rejuvenation has therefore the potential to be restored in function and improve the function of other aged organs at the same time. Here, we explore the evidence suggesting that the blood system plays a central role in overall tissue and organ aging and that its rejuvenation therefore improves health span.

### Role of hematopoietic cells in organismal aging and age‐related diseases

Hematopoietic cells have numerous roles, including molecular transport throughout the body, immune response, and body homeostasis. With time, these functions decline [124]. Here, we point out how hematopoietic cells relate to organismal aging and age‐related diseases:

*Blood system*—A declining blood system co‐occurs with additional disease risks:

- Patients with age‐related blood disorders display comorbidities that are listed in the top 10 causes of death by the World Health Organization: cardiac, renal, and pulmonary diseases, and solid tumor development [125, 126, 127, 128].
- An aging blood system often displays clonal hematopoiesis, wherein a subset of hematopoietic stem cell (HSC) clones acquires mutations increasing their proliferation thereby making up a large portion of the hematopoietic compartment. Clonal hematopoiesis is associated with increased risk of blood cancer, coronary heart disease and stroke and is associated with a 35% higher mortality risk [128] possibility via modulation of immune system function.
- An aging blood system is accompanied by increased risk of (pre‐)malignant hemopathies [129, 130, 131] and bone marrow fibrosis [132, 133, 134].
- Disease risk increases upon T‐cell decline: Increased risk of autoimmune diseases correlates with aging of T cells [135]. In patients with autoimmune disorders, such as rheumatoid arthritis, pro‐inflammatory T‐cell expansion drives tissue destruction, and promotes age‐related pathologies like cardiovascular disease [124]. T‐cell destruction upon HIV infection accelerates aging in AIDS patients, which also leads to cardiovascular disease, cancer, frailty, and osteoporosis [136, 137].
- The decline of red blood cells (RBC) results in anemia that affects about one‐third of the world population. Even mild anemia is associated with age‐associated diseases, such as cancer and renal insufficiency [138].

Overall, these observations reveal that a declining blood system correlates with dysfunction in other organs.

*Immune system*—During old age, a drastic drop in the efficiency of the immune cells and an accumulation of pro‐inflammatory cytokines and chemokines result in organismal decline. Indeed, transplanting senescent immune cells causes, (a) senescence in nonlymphoid tissues and solid organs, (b) loss of muscle regeneration, (c) organ damage, especially in the heart, liver, kidneys, and brain, and (d) reduced lifespan in recipient mice [81]. This organismal decline is expressed in various ways:

- *Infections* become more frequent and vaccination responsiveness decreases with age [124, 139].
- *Systemic inflammation* accelerates cellular and organ aging [140, 141]. T‐cell dysfunction increases systemic pro‐inflammatory cytokines, contributing to organ decline and reduced lifespan [81, 90, 124, 142, 143, 144]. For example, the expansion of CD8+ T cells in the spleen, peritoneum, liver, and lung produces the pro‐inflammatory Granzyme K, which has been proposed as a trigger of age‐driven inflammation [143]. Macrophages are also a main source of pro‐inflammatory cytokines and drive inflammation in the kidney and liver [145, 146]. Increased activation of neutrophils increases tissue inflammation, such as periodontitis, and contributes to age‐associated disease onset [147, 148].
- *Clearance activity* of cytotoxic T cells declines during aging, slowing down the removal of premalignant and senescent cells, which facilitates cancer and organismal decline [90, 149, 150]
- *Organ repair* decreases with declining immune function [151, 152]. For instance, lower abundance of neutrophils alters organ repair [153] and delays bone fracture healing [154].

Overall, these findings demonstrate that the hematopoietic system and especially the immune system play a fundamental role in organismal functions beyond the blood system and therefore are likely to be a key aspect of organismal aging [16].

### Blood system—A target for organismal rejuvenation

Several strategies have been explored to rejuvenate the blood system, which in turn further demonstrates its influence on the function of other organs:

- *Heterochronic parabiosis* is a procedure that merges the blood systems of old and young mice. It thereby increases rejuvenation markers and processes in many organs of the old animal while the young animal displays increased aging characteristics [155, 156, 157, 158]. The rejuvenation is likely caused by rejuvenating factors from the young circulatory system [159, 160, 161, 162, 163] and the dilution of pro‐aging factors from the aged compartment [164, 165, 166]. Supplying old animals with young blood cells improves senescent cell removal [157, 167, 168], bone repair [169, 170], and the regeneration of the central nervous system (CNS) [158, 160, 161, 171]. This procedure unveils the potential of the blood compartment as a central rejuvenation tool [155].
- *Therapeutic plasma exchange* (TPE) is a procedure in which a patient's plasma is removed and replaced with a substitute fluid like saline, albumin, or donor plasma. In old mice, plasma dilution promotes neurogenesis and rejuvenates skeletal muscles and the liver [164, 166]. In humans, TPE reduces the aging‐associated myeloid bias, systemic inflammation, DNA damage, and senescence in peripheral blood mononuclear cells [165]. TPE is currently used as a therapeutic strategy for numerous diseases [172, 173], but its potential to treat age‐related diseases remains to be explored.
- *RBC peri‐transfusion* is the main treatment for anemia or hemoglobinopathies. Several treatments have been developed to rejuvenate RBCs, thereby improving their capacities following hypothermic conservation [174, 175, 176, 177]. *In vivo*, these rejuvenated RBCs improved the oxygenation and function of the heart, lungs, and kidneys [178]. RBC rejuvenation could therefore be beneficial for aging individuals.
- *Transplantation* of bone marrow cells promotes functional recovery beyond the blood system by contributing to muscle regeneration [179], repair of heart muscle tissue [180], improving postnatal blood vessel formation [181], bone healing [169], and cognitive functions [182]. Notably, transplanting young bone marrow or progenitor‐enriched bone marrow (Lin‐ cells) into old mice significantly increased their lifespan by about 31% or 12%, respectively [183, 184]. The transplantation of cord blood cells, enriched for bone marrow cell types, attenuates the accelerated aging phenotype driven by progeria [185].

#### Restoring immune cell function

- Removing senescent T cells from adipose tissue improves glucose tolerance, insulin resistance, and obesity‐related metabolic disorders in mice [186]. Importantly, the detrimental effects driven by dysfunctional T cells are reversible in the heart, the visceral adipose tissue and other key organs [81, 142, 186, 187]. A promising way to counteract T‐cell‐driven aging is to restore thymus function [188, 189].
- Exercise‐induced rejuvenation of neutrophils co‐occurs with reduced disease risk in aged patients with type 2 diabetes predisposition [190].
- Heterochronic parabiosis suggests that monocytes of young animals have the potential to regenerate the CNS of old animals by supporting the process of building new myelin sheath [171].

Overall, these findings demonstrate that rejuvenation of hematopoietic cells restores functions beyond the blood system, crystallizing it as an optimal therapeutic target to prevent organismal aging.

## Hematopoietic stem cells—A promising rejuvenation target?

### Effect of time on HSCs

**Fig. 1:**
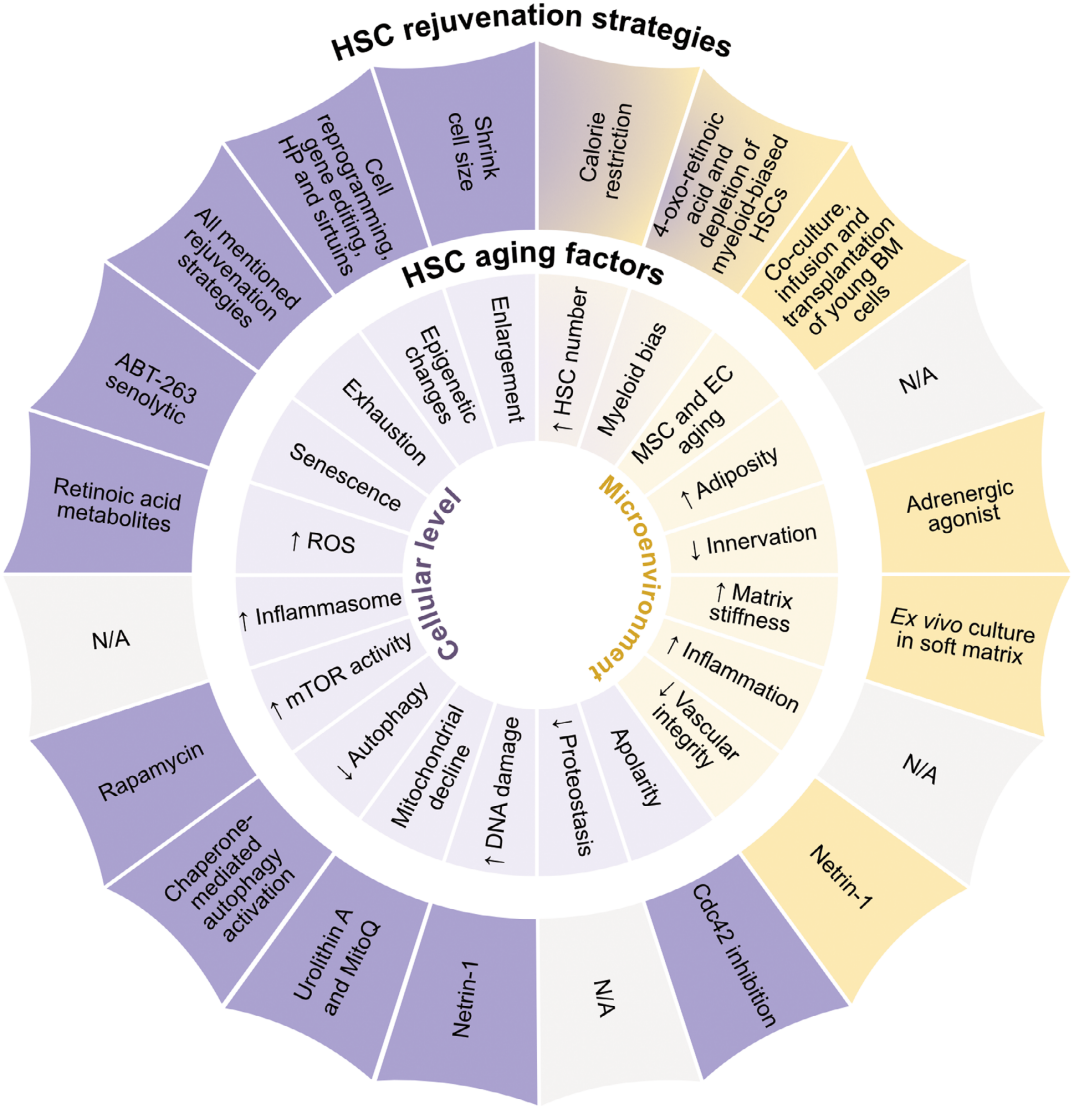
Hematopoietic stem cell (HSC) aging factors and rejuvenation strategies. This diagram represents the factors leading to HSC aging (inner circle), and the rejuvenation tools targeting these aging factors (outer circle). The aging drivers can originate from the HSCs themselves (purple) or the microenvironment (yellow), or a combination of both (purple and yellow). For some factors, no HSC‐specific rejuvenation strategy currently exists and is marked as N/A. See the main text for more details. HP, heterochronic parabiosis.

HSCs are at the top of the hematopoietic hierarchy, giving rise to all hematopoietic cells. During aging their stemness declines, affecting downstream hematopoietic cells like immune cells [191, 192]. To explore the potential to rejuvenate HSCs, we first need to understand the processes of aging and adaptations in HSCs (Fig. 1):

- HSCs decline in function with time [191, 193]. This is driven by intrinsic factors, such as enlargement [8], apolarity [194], metabolic changes [121, 195], reduced DNA damage repair [196, 197], low proteostasis [198], low mitochondrial function [199], declining autophagy [200], increased mTOR activity [201], increased inflammasome [202], increased ROS levels [203, 204], senescence [186], stem cell exhaustion [205], epigenetic changes [206, 207, 208, 209], and possibly transposable element expression [209, 210].
- While individual HSC function declines, the HSC pool increases in number over time in humans and mice [101, 211]. This expansion may be a compensatory effort to maintain overall productivity of the HSC compartment. However, this process is often associated with clonal hematopoiesis [128, 211, 212], making the blood system more prone to leukemia transformation [212, 213].
- Aged HSCs bias toward myeloid lineage production at the expense of lymphoid lineage [214, 215, 216, 217, 218]. This increases inflammation [219] and reduces the adaptive immune response [120, 220, 221], which is associated with decreased cancer immunosurveillance [222].
- HSCs are affected by their complex bone marrow microenvironment, which is called the niche [223, 224]. During aging, the niche undergoes alterations, including increased matrix stiffness, vascular remodeling, decreased innervation, increased adiposity, and inflammation, which contribute to the decline of HSC function [225, 226]. Indeed, an old recipient's microenvironment reduces the ability of young HSCs to engraft and produce T cells. Inversely, transplanting old HSCs into young recipients results in more balanced myeloid/lymphoid lineages [227, 228]. However, a young niche is not sufficient to restore the function of old HSCs [229].

Interestingly, aging does not uniformly affect the HSC population, creating subsets of differently aged HSCs [120, 121, 122, 211, 230]. Overall, aging and the resulting adaptations progressively impair the ability of HSCs to ensure the functionality of the blood system.

### Rejuvenation strategies for HSCs and their effect on organismal functions

Transplanting young HSCs into aged mice significantly extends their health‐ and lifespan [122, 183], which indicates that the rejuvenation of old HSCs could have similar effects. In this part, we present strategies that restore the function of old HSCs [231] and present the effect on organismal functions. These studies analyzed the rejuvenation of HSCs at different levels: (a) HSC cellular characteristics like DNA damage, (b) *in vivo* HSC function, like engraftment, blood‐building capacity and lineage bias analyzed after transplantation into untreated recipient mice, overcoming pleiotropic effects, and (c) health‐ and lifespan of HSC recipients.

1. Rejuvenating old HSCs by restoring intrinsic pathways
  - Preventing or reversing age‐related HSC enlargement improves their blood‐building capacity upon transplantation [8, 10].
  - Rapamycin inhibits mTOR, extends lifespan [232], and rejuvenates old HSCs as their transplantation improves blood‐building capacity and lineage balance [8, 201]. Rapamycin also improves HSC function when administered *in vitro*, demonstrating its direct effect [233].
  - Inhibition of RhoGTPase Cdc42 with CASIN in aged mice partially rejuvenates aged HSCs by restoring their apolarity and improves their capacity to build immunocompetent cells. Remarkably, transplanting these rejuvenated HSCs increases the lifespan of aged immunocompromised recipient mice [192, 234]. Increased activity of Cdc42 is also associated with aging in humans and aged HSCs [235, 236, 237, 238].
  - Converting aged HSCs into induced pluripotent stem cells (iPSCs) by expressing the Yamanaka factors and then differentiating these back into HSCs effectively rejuvenates them transcriptionally and improves their blood‐building capacity and T‐cell function [120, 239].
  - Sirtuins are NAD+‐dependent deacetylases implicated in inflammation, metabolism, and oxidative stress response [240]. Sirtuins were established as promising pro‐longevity genes [241], although this is also disputed [242]. In mouse HSCs, overexpression of SIRT2/3/7 improves blood‐building capacity after transplantation, and SIRT2/7 improves lineage balance [202, 243, 244].
  - Mitophagy induction by *in vitro* or *in vivo* Urolithin A treatment or the restoration of mitochondrial membrane potential with MitoQ improves the capacity of old HSCs to build blood after transplantation [245, 246]. Old mice supplemented with Urolithin A also show an improvement in their immune response after an acute viral infection.
  - Activation of chaperone‐mediated autophagy *in vitro* or *in vivo* improves the function of old HSCs [247]. This is evidenced by enhanced long‐term self‐renewal capacity of aged HSCs *in vitro*, increased GAPDH activity and decreased protein oxidation level.
  - Modulating the expression of age‐associated genes, such as p38 MAPK, Satb1, Per2, Phf6, and Rantes/Ccl5, partially rejuvenates old HSCs. These approaches improve HSC commitment toward the lymphoid lineage. In addition, p38 MAPK inhibition and Phf6 deletion improve long‐term blood reconstitution. Of note, Per2−/− aged mice present an improved immune function and lifespan, although this may not be solely caused by rejuvenated HSCs because the gene is deleted in all cell types [227, 248, 249, 250, 251, 252, 253]
2. Rejuvenating old HSCs by systemic interventions
  - Aged HSCs contribute to the generation of pro‐inflammatory myeloid cells, which infiltrate cardiac tissue after myocardial infarction. Enforcing HSC quiescence with 4‐oxo‐retinoic acid, a vitamin A metabolite, mitigates inflammatory myelopoiesis, thereby improving tissue remodeling and preserving long‐term cardiac function [254, 255].
  - In middle‐aged mice, long‐term calorie restriction (CR) shows positive and negative effects [256]: It limits the increase of the HSC pool observed upon aging, and overall improves their self‐renewal and repopulation capacity upon transplantation. However, long‐term CR specifically inhibits the proliferation of lymphoid progenitors, resulting in an impaired immune function. In old mice, life‐long CR resulted in opposite results upon transplantation with either no impact on HSC function [257, 258] or improving the blood‐building capacity and maintaining the lymphoid/myeloid balance [259, 260].
  - Heterochronic parabiosis rejuvenates the expression profile of HSCs from old mice [156, 157] and may restore the lineage bias after transplantation [257, 261], while it fails to restore their blood reconstitution capacity [257].
  - Strategies that are commonly used to drive rejuvenation in other cell types like TPE and exercise do not seem to rejuvenate old HSCs [257].
3. Removing old HSCs
  - Clearing senescent cells, either from niche or HSCs themselves, rejuvenates the remaining HSCs in aged mice, and improves their ability to build a new blood system [262].
  - Depleting myeloid‐biased HSCs restores balanced differentiation in aged mice [220]. This depletion results in more lymphocyte progenitors and naive T and B cells. It improves adaptive immune responses, while decreasing age‐related markers of immune decline.
4. Targeting the niche
  - The infusion of young bone marrow‐resident endothelial cells in old mice rejuvenates HSC function and improves their engraftment and blood‐building capacity [263].
  - Supplementing old mice with niche‐derived factors like netrin‐1 [264] or with adrenergic agonists to stimulate the sympathetic system [265, 266] rejuvenates their niche, demonstrated by improved bone marrow vascular integrity, mesenchymal stem cell (MSC) number, and lower DNA damage levels. These approaches also improve blood‐building capacity of HSCs.
  - Softening the extracellular matrix stiffness rejuvenates old HSCs *ex vivo* and restores their blood reconstitution capacity, lineage balance, mitochondrial function, cell polarity, and DNA damage level [267]. Importantly, this approach requires the support of bone marrow‐resident MSCs, implying a functional connection between HSCs and MSCs (details below).
  - However, a young niche is not sufficient to fully restore the function of old HSCs [229], which implies that both intrinsic and extrinsic aging factors have to be targeted to optimize HSC rejuvenation.

We note that most of these studies were conducted in mice, and for most treatments, it is unclear whether they are also promising for human HSCs. The murine and human blood systems exhibit both similarities and differences when comparing aging hallmarks [268]. For instance, clonal hematopoiesis occurs more frequently in humans than in mice [269]. Thus, there is a need to expand aging research on human HSCs.

Overall, HSC rejuvenation can be achieved by multiple approaches and has far‐reaching effects like protecting immune functions and increasing lifespan. HSCs and their niche are therefore promising targets to improve health span.

## Mesenchymal stem cells—Support for blood system rejuvenation

In the bone marrow, HSCs are surrounded by niche cell types, including MSCs [270]. MSCs influence the function of hematopoietic cells [271]. For example, the co‐transplantation of HSCs with MSCs improves HSC engraftment, short and long‐term reconstitution and accelerates lymphocyte recovery [272, 273, 274, 275]. Co‐culturing of HSCs with young MSCs or HSC niche factors enhances the HSC's ability to build a blood system *in vivo* [276, 277]. In addition, MSCs modulate immune responses by, for example, limiting T‐cell pro‐inflammatory activity [278, 279, 280, 281, 282, 283, 284, 285, 286, 287, 288, 289, 290, 291, 292], which can mitigate the graft‐versus‐host disease upon co‐transplantation with HSCs [293, 294, 295]. In turn, HSCs improve the function of damaged MSCs [296]. Hence, these two cell types influence each other's rejuvenation capacity.

Upon aging, senescent MSCs alter immune cells thereby impairing bone and cardiac regeneration and driving organ inflammation [145, 146, 297, 298, 299, 300]. Impaired MSCs also promote tumorigenesis, myeloproliferative diseases and bone marrow fibrosis [133, 301, 302, 303, 304, 305]. Rejuvenating or removing senescent MSCs restores their immunoregulatory activity and improves bone regeneration, angiogenesis and cardioprotection following infarction [297, 300, 306]. The infusion of young MSCs restores immunomodulatory activities and reduces tissue deterioration driven by autoimmune disease [307, 308]. In line with this, several clinical studies use MSCs for skin regeneration and to treat neurodegenerative and ischemic heart diseases [309, 310, 311]. Thus, targeting MSCs is a promising approach for the rejuvenation of immune cells and organs. Interestingly, the organs affected by MSC‐based cell therapies are similar to the ones for HSC‐based therapies (see below), which further strengthens the functional interplay between MSCs and HSCs in rejuvenation potential. Unfortunately, the advantages of MSC‐based therapy in patients with autoimmune disease are inconsistent and often lost in the long term, which implies a short‐term maintenance of MSCs in recipients and the need for repeated infusions [312, 313, 314, 315, 316, 317, 318, 319, 320, 321, 322, 323, 324]. However, these findings place MSCs as a promising partner to the hematopoietic system for immune cell and organ rejuvenation.

## HSC‐derived (immuno)therapies to counteract aging‐related disease

HSC transplantation is the main stem cell‐based therapy in humans and is used for treating diseases of the blood system, like hematological malignancies and autoimmune diseases [325, 326]. In patients with autoimmune diseases, for example, autologous HSC (aHSC) transplantations reset the T‐cell repertoire, which improves immune cell function and disease outcomes [327, 328, 329, 330, 331, 332, 333, 334, 335, 336]. Furthermore, several clinical studies have evaluated the potential of HSC transplants to treat nonhematopoietic diseases:

- *Solid tumors*: HSC transplants can generate an immune response, called graft‐vs‐tumor effect, improving the survival of patients by the elimination of cancerous cells [337, 338, 339].
- *Angiogenesis and organ arterial blood supply uponischemia*: HSC transplants may promote angiogenesis by generating new endothelial cells [340].
- *Neurological disorders*: HSC transplants improve brain repair potential in patients with neurological disorders [341, 342]. Together with HSC gene therapy, HSC transplants are an efficient treatment for patients with cerebral adrenoleukodystrophy and metachromatic leukodystrophy [343, 344, 345, 346, 347].
- *Skin disorder*: HSC transplants improve wound healing and reepithelialization of the skin in epidermolysis bullosa patients and diabetic mice [348, 349].
- *Systemic sclerosis*: HSC transplants decrease all‐cause mortality and improve lung capacity and skin thickness [330].

An interesting question is how aHSC transplants improve nonhematopoietic diseases. One supported model is that they restore a functional immune system, which in turn improves the health of other organs by, for example, removing premalignant and senescent cells as outlined above [81, 90, 124, 142, 144]. Another model proposes that HSCs transdifferentiate into nonhematopoietic cell types [350, 351, 352, 353, 354, 355, 356]. However, HSCs rarely generate nonhematopoietic cell types during physiological conditions in mice [357]. Lastly, hematopoietic cells may also improve cardiac, neuronal and hepatic functions via cell fusion [358]. Altogether, even though the safety of the procedure has to be improved [348, 359, 360], aHSC transplants reveal a new perspective on how to counteract certain aging‐related diseases.

HSCs are also interesting for approaches using engineered chimeric antigen receptors (CARs). CARs enable T and NK cells to recognize specific antigens and to target, for example, cancer cells [361, 362]. This approach is also utilized to target fibrotic and senescent cells, thereby preserving the integrity of cardiac and liver tissue following injury [363, 364, 365]. However, the high costs of CAR T‐cell engineering and their short maintenance in recipients motivate the search for alternatives [361]. HSCs display long‐term self‐renewal capacities and multipotency. Hence, engineering HSCs for immunotherapies allows for long‐lasting and diverse replenishment of chimeric immune cells [366, 367, 368]. For instance, HSC engineering can overcome the resistance of NK cells to viral transduction and generate HSC‐derived invariant NK cells for cancer immunotherapies [362, 369, 370]. The potential of HSCs for immunotherapies is further enhanced by the outcome of clinical studies on hematological and autoimmune disorders; up to 15 years follow‐up on subjects confirmed the long‐term biological safety and efficacy of gene therapy using lentivirally transduced HSCs [368, 371, 372, 373, 374, 375, 376, 377, 378, 379]. Excitingly, the development of nanoparticles and viral vectors might even enable *in vivo* editing of HSCs [380, 381, 382, 383, 384, 385, 386]. Altogether, autologous transplantation of rejuvenated and engineered HSCs is a promising tool to slow down age‐related disease occurrence.

## Conclusion

The current interest in anti‐aging and rejuvenation strategies motivated us to discuss the importance of understanding lifespan and aging before interfering with them. We find compelling arguments that the long and healthy lifespan of humans provides benefits from enabling complex brain development to building efficient social structures of intergenerational care supporting survival and reproduction.

Aging is a complex process manifesting itself differently across tissues and cell type populations. Importantly, it is still not fully understood. To establish successful rejuvenation strategies, it is essential that we improve our understanding of the holistic picture of all factors driving aging and their interactions.

The blood system, with HSCs at the top of its hierarchy, appears to play a central role in organismal aging. HSC aging is driven by intrinsic mechanisms and the bone marrow microenvironment. It impacts hematopoiesis with consequences within and beyond the blood system, contributing to organismal aging. Thus, restoring a functional blood system by rejuvenating HSCs is expected to also improve the function of other organs thereby reducing the risk of developing a broad range of age‐associated diseases. While HSC rejuvenation is not expected to benefit all organs and tissues, such as the endocrine system, future work will show whether HSC‐derived improvements are enough to counteract overall decline at old age.

Techniques to rejuvenate the blood compartment are expanding and improving. Autologous HSC transplantations in humans further illustrate what organismal rejuvenation strategies can achieve as they are already used to treat hematopoietic and nonhematopoietic diseases. The supplementation of MSCs or other niche factors might enable HSCs to reach their full rejuvenation potential. Overall, our review highlights the power of the hematopoietic compartment to reverse organismal aging.
